# Clinical Association of Chemokine (C-X-C motif) Ligand 1 (CXCL1) with Interstitial Pneumonia with Autoimmune Features (IPAF)

**DOI:** 10.1038/srep38949

**Published:** 2016-12-13

**Authors:** Minrui Liang, Zhixing Jiang, Qiong Huang, Lei Liu, Yu Xue, Xiaoxia Zhu, Yiyun Yu, Weiguo Wan, Haihua Yang, Hejian Zou

**Affiliations:** 1 Division of Rheumatology, Huashan Hospital, Fudan University, Shanghai 200040, P.R. China; 2 Institute of Rheumatology, Immunology and Allergy, Fudan University, Shanghai 200040, P.R. China; 3 Department of Dermatology, Huashan Hospital, Fudan University, Shanghai 200040, P.R. China; 4 Department of Pulmonology, Huashan Hospital, Fudan University, Shanghai 200040, P.R. China

## Abstract

The term “interstitial pneumonia with autoimmune features” (IPAF) has been recently proposed. We here investigate the clinical characteristics of IPAF and evaluate the clinical implications of CXCL1-CXCR2 axis in IPAF. An increased plasma level of CXCL1 was exhibited in IPAF compared to idiopathic interstitial pneumonia (IIP), chronic obstructive pulmonary disease (COPD), and healthy controls. Additionally, plasma CXCL1 levels were clinically associated with diffusing capacity of the lungs for carbon monoxide (DLCO), erythrocyte sedimentation rate (ESR), and involved parenchyma extension in IPAF. Furthermore, circulating CXCL1 levels were highest in IPAF patients with acute exacerbations. CXCR2, the chemokine receptor for CXCL1, was readily observed in inflammatory aggregates and endothelial cells in IPAF lungs, but was lower in IIP lungs and healthy lungs. Interestingly, increased CXCL1 concentrations in BALF paralleled neutrophil counts in IPAF. Overall, the plasma concentrations of CXCL1 indicated the disease activity and prognosis in IPAF. Thus, the CXCL1/CXCR2 axis appears to be involved in the progression of IPAF.

Interstitial lung disease (ILD) represents a highly heterogeneous group of diseases. Generally, ILD has no identifiable underlying cause and is regarded as idiopathic, as in idiopathic interstitial pneumonia (IIP). However, ILD can be associated with a specific environmental exposure or underlying connective tissue disease (CTD). As such, ILD patients with overlapping features of both ILD and systemic autoimmune disorders that do not meet any defined CTD criteria constitute a grey zone for both rheumatologists and pulmonologists in clinical practice. In July 2015, the European Respiratory Society (ERS)/American Thoracic Society (ATS) jointly proposed a novel entity termed “interstitial pneumonia with autoimmune features” (IPAF) to describe individuals with both ILD and combinations of other clinical, serologic, and/or pulmonary morphologic features, which putatively stem from an underlying systemic autoimmune condition but do not meet current rheumatologic criteria for a characterized CTD^1^.

We focused on individuals with concomitant interstitial pneumonia and autoimmune features that fulfilled the recently proposed classification criteria for IPAF^1^, their clinico-immunologic characteristics were analysed and compared with a series of individuals with IIP. In particular, we hypothesized that patients with IPAF, who have a potential underlying autoimmune condition, may have a different inflammatory and immunologic profile than patients with IIP. Moreover, clinico-immunologic monitoring of patients with IPAF may allow for the investigation of specific pathogenic mechanisms as well as potential parameters with which to evaluate disease severity and predict progression. Distinguishing those who develop severe disease from those who develop slow or stable disease remains a great challenge in targeting appropriate therapy.

In pulmonary inflammation, the recruitment of circulating leukocytes is essential for host defence and initiates the specific immune response. The released chemokines from the site of inflammation induce the extravasation of leukocytes from the vascular system into the tissue. CXCR2 is of particular interest because several studies have implicated a pivotal role of this receptor in the development and promotion of numerous inflammatory disorders. CXCR2 is a 7-transmembrane G protein-coupled receptor that is activated by CXC chemokines containing the ELR (Glu-Leu-Arg) motif, including CXCL1^2^. CXCL1 plays an important role in inflammation, angiogenesis, tumourigenesis, and tissue healing^3^,^4^,^5^,^6^,^7^,^8^,^9^. The CXCL1-CXCR2 axis is activated in many lung diseases^3^,^4^,^5^,^6^,^7^,^8^,^9^, and the levels of this chemokine are often correlated with the clinical activity of these diseases and poor prognosis^10^,^11^,^12^. Modulation of the function of CXCR2 is therefore considered to be a potential therapeutic strategy in the treatment of inflammatory conditions in humans^13^,^14^,^15^,^16^,^17^.

The present study was undertaken to describe the clinico-immunological features of IPAF and gain insight into the potential hallmarks in the observed patients with IPAF. We also compared the clinico-immunological features of the IPAF patients with the IIP patients enrolled in this study over the same period. Furthermore, we hypothesized that CXCL1 may be increased and clinically associated in patients with IPAF. If so, these findings would help to substantiate the role of the CXCL1-CXCR2 axis in IPAF, potentially identify a useful biomarker for evaluating disease severity and predicting disease progression in this population, and support the rationale for CXCL1/CXCR2-targeted treatments in these patients.

## Results

### Subjects

The characteristics of the lung disease subjects who provided phlebotomy specimens for plasma cytokine assays are detailed in Table 1. The average age of the IPAF patients was 56 ± 2.4 years (yr), which was significantly lower than the ages of IIP patients (65 ± 1.7 yr, P = 0.0019, IPAF *vs* IIP) or COPD (70 ± 1.8 yr, P < 0.0001, IPAF *vs* COPD) patients (Table 1). Among the IPAF patients, the percentage of men and women was almost identical, and the proportion of males among both IIP patients and COPD patients was much greater than among IPAF patients (P = 0.2289, IPAF *vs* IIP; P = 0.019, IPAF *vs* COPD) (Table 1).

The demographic and clinical characteristics of the lung disease subjects who underwent a fiberbronchoscopy examination and provided bronchoalveolar lavage fluid (BALF) for cytokine assays are summarized in Supplementary Table 1. The age of IPAF patients (49 ± 6.9 yr) was significantly lower than that of IIP patients (67 ± 3.1 yr, P = 0.0447, IPAF *vs* IIP) (Supplementary Table 1). The percentages of males and females in IPAF patients were equivalent. A greater proportion of IPAF patients were male (80%) compared with the IPAF patients (50%) (P = 0.004) (Supplementary Table 1).

**Figure 1:**
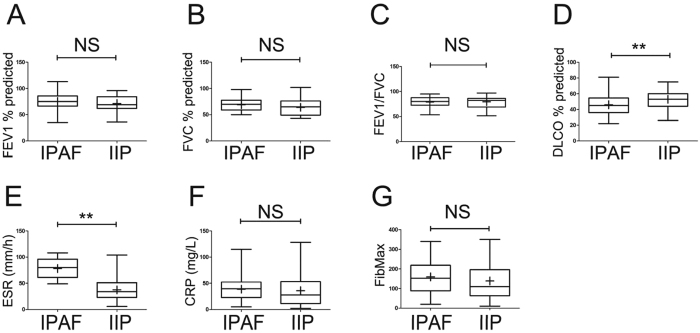
Analyses of the clinical parameters of IPAF patients and comparisons with those of IIPpatients. ESR (D) and DLCO (E) were exclusively significantly higher in the subjects with IPAF than in those with IIP. The horizontal line from bottom to top denotes the minimum, 25th percentile, median, 75th percentile, and maximum. Mean values are denoted by “+”. *P < 0.05 and **P < 0.01 compared with the other cohort.

Herein, we further compared the differences between IPAF and IIP for the other clinical parameters. The pulmonary function test results demonstrated restrictive ventilation dysfunction in both IPAF and IIP patients, which were completely different from the obstructive ventilation dysfunction in COPD patients (Table 1). Furthermore, there were no significant differences in forced vital capacity (FVC), forced expiratory volume in 1 s (FEV1), or FEV1/FVC between IPAF patients and IIP patients (Table 1 and Fig. 1A,B,C). However, the value of DLCO in IPAF patients was significantly lower than that in IIP patients (P = 0.0048) (Table 1 and Fig. 1D), which indicated that the reduced pulmonary diffusing capacity in IPAF was much more severe than that in IIP. The erythrocyte sedimentation rate (ESR) was elevated in IPAF (78.2 ± 3.2, mm/h) and was much higher than that in IIP (37.4 ± 2.3, mm/h) (P < 0.0001, Fig. 1E). In contrast, the C-reactive protein (CRP) levels were similarly increased in IPAF and IIP (P = 0.5896) (Fig. 1F). The extension of the involved lung parenchyma determined by FibMax was higher in IPAF (159.6 ± 14.3) than that in IIP (138.6 ± 10.6) but did not reach statistical significance (P = 0.2521) (Fig. 1G).

### Cross-sectional Assays of circulating cytokines

**Figure 2:**
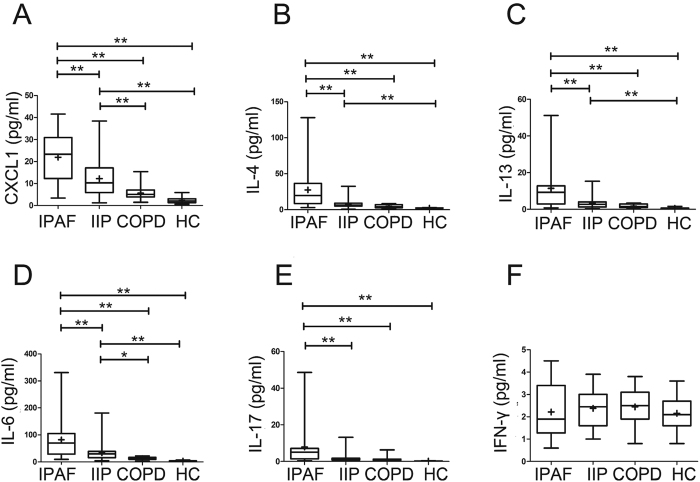
Concentrations of circulating CXCL1 (**A**) IL-4 (**B**) IL-13 (**C**) IL-6 (**D**) IL-17 (**E**) and IFN-ã (**F**) in the plasma of lung disease subjects using a multiplex Luminex immunoassay. The horizontal line from bottom to top denotes the minimum, 25th percentile, median, 75th percentile, and maximum. Mean values are denoted by “+”. *P < 0.05 and **P < 0.01 compared with the other cohort.

The concentrations of CXCL1, IL-4, IL-13, IL-6 and IL-17 in the plasma were significantly higher in patients with IPAF compared with IIP and the normal cohorts, but no significant difference in the plasma levels of interferon-γ (IFN-γ) was found in the patients with IPAF, IIP, COPD and healthy controls (Fig. 2). The plasma levels of CXCL1, interleukin-4 (IL-4), IL-13 and IL-6 in IIP patients were also higher than those in healthy controls (Fig. 2). In addition to CXCL1, CXCR2 binds to chemokines of the CXC family containing the glutamate-leucine-arginine (ELR) motif (i.e., CXCL2, CXCL5 and CXCL8 in human)^18^. Similarly, the plasma concentrations of CXCL2, 5 and 8 were relatively higher in IPAF patients compared to those in IIP patients. Only the plasma CXCL8 level in IPAF patients was significantly different from IIP patients (Supplementary Fig. 1). Sex and age had no discernible effects on plasma CXCL1, IL-4, IL-13, IL-6, IL-17, CXCL2, CXCL5, and CXCL8 concentrations among normal control subjects or subjects with IPAF or IIP (data not shown).

### Clinical correlations of CXCL1 in IPAF

Given the findings here that circulating CXCL1, IL-4, IL-13, IL-6, IL-17, CXCL2, CXCL5, and CXCL8 were abnormally increased in the IPAF cohorts (Fig. 2), we examined the plasma concentrations of these cytokines for associations with the clinical features of the subjects with IPAF.

**Figure 3:**
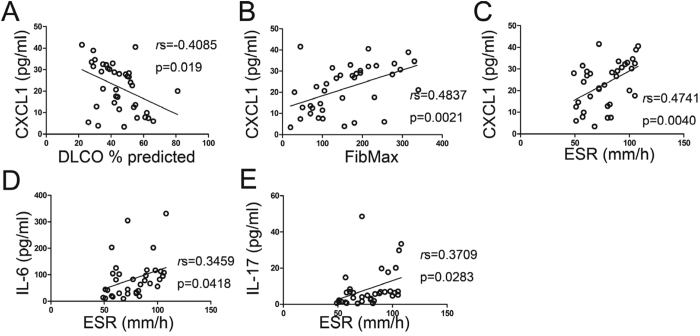
Clinical correlations of circulating cytokine levels in IPAF patients.

CXCL1 levels among the subjects with IPAF were inversely associated with DLCO (Fig. 3A), whereas CXCL1 was positively correlated with FibMax scores and ESR levels in subjects with IPAF (Fig. 3B and C). The plasma concentrations of IL-6 or IL-17 were positively correlated with ESR levels in the subjects with IPAF (Fig. 3D and E). No other correlation was found between the plasma cytokine levels with the clinical parameters in the subjects with IPAF. However, in the subjects with IIP, only the correlations of IL-4, IL-13 or IL-17 with FibMax scores were found (Supplementary Fig. 2).

**Figure 4:**
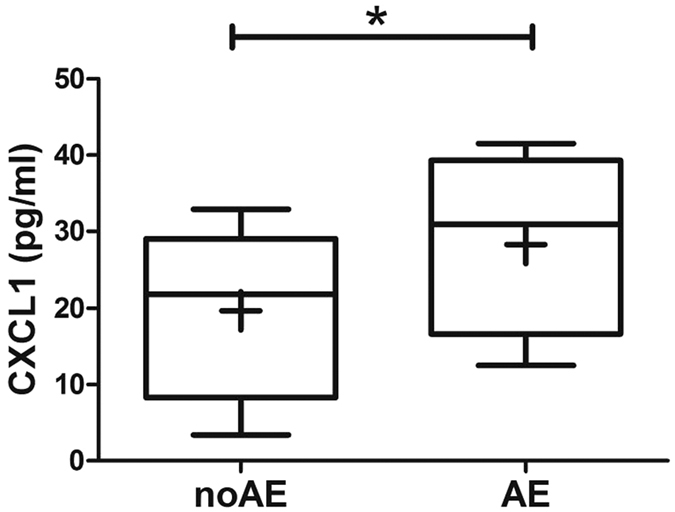
CXCL1 concentrations were the highest in patients with IPAF who were experiencing, or expected to experience, acute exacerbations (AEs) in the next 6 months (n = 6). The horizontal line from bottom to top denotes the minimum, 25th percentile, median, 75th percentile, and maximum. Mean values are denoted by “+”. *P < 0.05 and **P < 0.01 compared with the other cohort.

Because CXCL1 has been implicated as a major chemokine that binds to the CXCR2 receptor and drives neutrophil recruitment, leading to lung injury^5^, we evaluated the potential prognostic role of CXCL1 for patients with acute exacerbation (AE) of IPAF. Ten of 38 patients with IPAF in this cohort, who were experiencing AE at presentation or would to have AE within the next 6 months, also had significantly greater concentrations of CXCL1 in their plasmas (plasma CXCL1 in non-AE: 19.7 ± 2.0 ng/ml; plasma CXCL1 in AE: 28.3 ± 3.5 ng/ml; P = 0.0347) (Fig. 4).

We next assessed the role of CXCL1 as a potential clinical indicator for IPAF and stratified the patients with IPAF into the quartile with the highest circulating CXCL1 levels compared with the remaining 75% of subjects with lower levels of CXCL1. There was no difference in terms of age between the highest and lowest CXCL1 groups (Table 2). A preponderance of male subjects was observed in the highest CXCL1 group, which was different from the equal proportion of males and females in the lowest CXCL1 group but did not reach statistical significance (P = 0.7256) (Table 2). The pulmonary function test results demonstrated similar levels of FVC, FEV1 and FEV1/FVC in the lowest and highest CXCL1 subpopulations. Nevertheless, DLCO in the highest CXCL1 quartile was much lower than that in the lowest CXCL1 quartile (P = 0.0003). In addition, FibMax scores were much higher in the highest CXCL1 group than in the lowest CXCL1 group (P = 0.0069) (Table 2). Furthermore, the levels of ESR were significantly higher in the highest CXCL1 subgroup than that in the lowest CXCL1 subgroup (P = 0.0023) (Table 2).

### Intrapulmonary CXCR2

**Figure 5:**
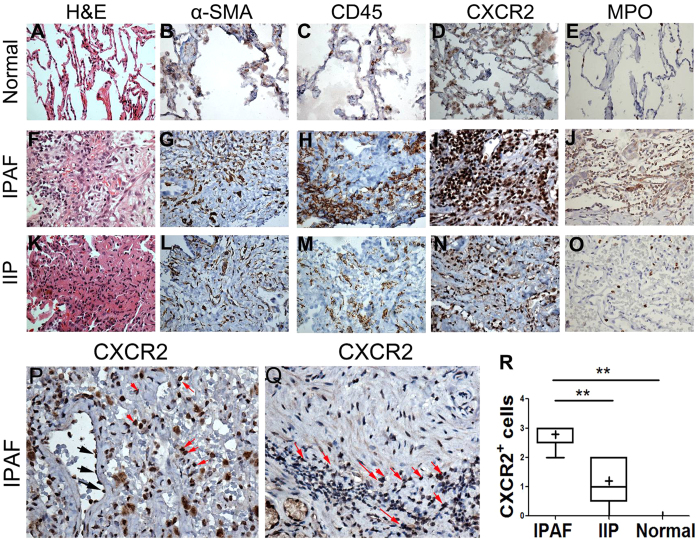
Immunohistochemical analyses of á-SMA, CD45, CXCR2, and MPO (**A**–**Q**). Lung samples from 5 patients with IPAF, 5 patients with IIP, and 3 normal controls were analysed and scored from 0 (no staining) to 3+ (bright and/or diffuse) (**R**). The horizontal line from bottom to top denotes the minimum, 25th percentile, median, 75th percentile, and maximum. Mean values are denoted by “+”. *P < 0.05 and **P < 0.01 compared with the other cohort. (**A**–**Q** For original magnification×400).

Because CXCL1 was abnormally increased in IPAF patients with significant clinical correlations, to substantiate the role of CXCL1 in IPAF, the expression and distribution of its cognate receptor CXCR2 was also examined in the lung needle biopsies from the patients with IPAF (n = 5), IIP (n = 5), and normal controls (n = 3). Although IPAF was differentiated from IIP because of the autoimmune tendency, we observed that IPAF and IIP may share similar histopathological patterns. The histological pattern of non-specific interstitial pneumonia (NSIP) was found in 3/5 cases of IPAF lungs, and organizing pneumonia (OP) was found in 2/5 cases of IPAF lungs. The histological pattern of NSIP was found in 2/5 cases of IIP lungs, and OP was found in 3/5 cases of IIP lungs. Activated fibroblasts within fibrotic foci were identified by their characteristic spindle-shaped morphology, demonstrating strong anti-α-SMA immunoreactivity on serial sections. We found that CD45^+^ cells (represented as leukocytes) were typically near α-SMA^+^ fibroblast foci in the lung biopsies of IPAF patients (Supplementary Fig. 3), which may indicate the close association of inflammatory infiltration with fibrotic process in the lungs of IPAF patients. The expression of CD45 detected by immunohistochemistry was strong and extensive in IPAF lungs (Fig. 5H). In contrast, the CD45 immunoreactivity was not remarkable in the specimens from patients with IIP (Fig. 5M). Moreover, serial sections of IIP lungs demonstrated that CD45^+^ aggregates were not typically distributed close to α-SMA^+^ fibrotic foci (Fig. 5L and M). Fewer CD45^+^ cells and α-SMA+ fibroblasts were detectable in the normal lungs (Fig. 5B and C).

Immunohistochemical staining of normal lung tissue demonstrated CXCR2 expression only in rare inflammatory cells, endothelial cells in the vessel walls, and occasional pneumocytes (Fig. 5D). In contrast, CXCR2 immunostaining was readily observed in the loosely formed lymphoid aggregates of IPAF lungs (Fig. 5I) but scattered in IIP lungs (Fig. 5N) and rare in normal lungs (Fig. 5D). IPAF lung needle biopsies demonstrated an increased presence of CXCR2^+^ cells proximate to fibrotic areas of lung parenchyma (Fig. 5Q, red arrows). Many CXCR2^+^ cells in inflammatory infiltration could be recognized as neutrophils (Fig. 5P, red arrows). Strong anti-CXCR2 immunoreactivity was also observed on the endothelial cells of IPAF and IIP lungs (Fig. 5I,N,P,Q; black arrows in 5 P). Pneumocytes stained positively for CXCR2 in IPAF and IIP (Fig. 5I,N,P,Q). Lung samples from 5 patients with IPAF, 5 patients with IIP, and 3 normal controls were analysed and scored from 0 (no staining) to 3+ (bright and/or diffuse). Samples from the patients with IPAF had stronger CXCR2 staining than did those from IIP (mean ± SE 2.80 ± 0.20 *vs* 1.20 ± 0.37; P = 0.0055) and normal controls (mean ± SE 2.80 ± 0.20 *vs* 0.67 ± 0.33; P = 0.0010) (Fig. 5R). An isotype control for CXCR2 staining confirmed the specificity of the signal (data not shown). An intense distribution of neutrophil labelling with myeloperoxidase (MPO) was found in the inflammatory infiltration of IPAF lungs (Fig. 5J), in which MPO^+^ staining was much more extensive relative to that in IIP lungs (Fig. 5O), and much fewer neutrophils were detected in normal lungs (Fig. 5E).

### Neutrophilic inflammation and CXCL1 upregulation in the BALF of IPAF

**Figure 6:**
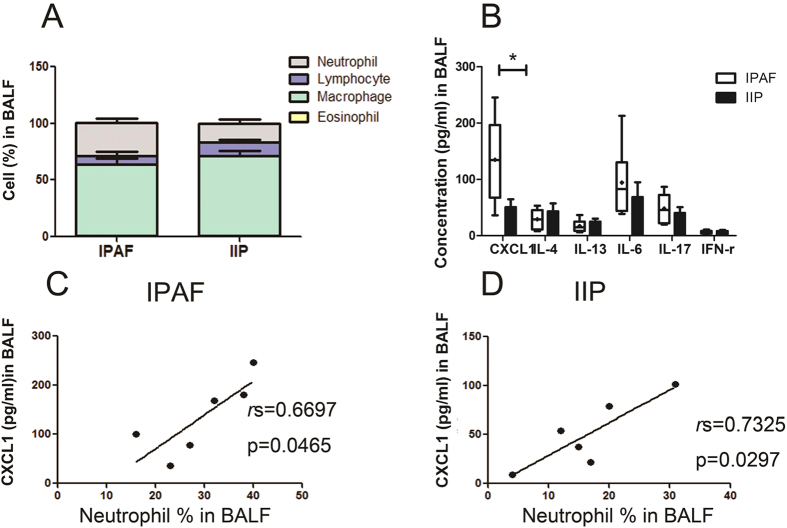
Cell percentages and cytokine concentrations in lung disease subjects. BALF specimens were available from subjects with IPAF (n = 6) and IIP (n = 6). The horizontal line from bottom to top denotes the minimum, 25th percentile, median, 75th percentile, and maximum. Mean values are denoted by “+”. *P < 0.05 and **P < 0.01 compared with the other cohort.

The percentage of neutrophils in IPAF BALF was remarkably greater than that in IIP BALF (IPAF: 29.3 ± 3.7%; IIP: 16.5 ± 3.7%; P = 0.0341) (Supplementary Table 2 and Fig. 6A). Macrophages were the predominant leukocyte type in the BALF of both IPAF and IIP patients; however, no difference was found in BALF proportions of macrophages, lymphocytes, and eosinophils between IPAF and IIP patients (Supplementary Table 2 and Fig. 6A). Significant elevations of CXCL1, 2, 5, and 8 in IPAF BALF were observed compared to those in IIP BALF (Fig.6B and Supplementary Fig. 4). However, no remarkable changes in the concentrations of IL-4, IL-13, IL-17 or IFN-γ were found in IPAF BALF relative to those in IIP BALF (Fig. 6B). The concentration of CXCL1 was shown to be closely correlated with the percentage of neutrophils in the BALF of both IPAF (Fig. 6C) and IIP (Fig. 6D). Also, there were correlations between BALF CXCL2, 5, and 8 levels with the percentage of neutrophils in BALF of IPAF and IIP patients, but without statistical significance (data not shown).

## Discussion

The data presented here suggest the presence of a different circulating cytokine profile in IPAF compared to IIP. In particular, we found that the patients with IPAF had abnormally increased levels of CXCL1 (Fig. 2A), which was highly associated with the clinical manifestations and disease severity in individuals with IPAF. Moreover, the CXCL1 level was greater and more clinically correlated in patients with IPAF than among lung disease control subjects with IIP or COPD, further indicating that the production of this chemokine is not only the consequence of lung injury but is specifically involved in the progression of IPAF.

CXCL1 is thought to exert its effects via the membrane CXCR2, by recruiting leukocytes, especially neutrophils, to the local sites, and by contributing to inflammation^2^. Our observation showed upregulated CXCL1 concentrations were associated with the BALF neutrophilia in IPAF and IIP patients (Fig. 6C and D), which may indicate the ability of CXCL1 to promote neutrophil chemotaxis within the alveolar compartment in both IPAF and IIP. Our findings in subjects with IPAF and IIP parallel analogous observations in patients with other disease conditions in which the accumulation of neutrophils was shown to be driven by CXCL1^3^,^4^,^5^,^19^.

Remarkably, the assays here showed that CD45^+^ leukocytes were near the fibroproliferative lesions in IPAF lungs (Supplementary Fig. 3), whereas this typical phenomenon was not seen in IIP lungs (Fig. 5L and M). CXCR2^+^ cells, in turn, were found within pulmonary leukocyte aggregates in the patients with IPAF (Fig. 5I,P, and Q). Neutrophils, readily identified by the expression of MPO, are the predominant CXCR2+ cells among blood leukocytes, and CXCR2 is a key regulator of their recruitment and effecting responses^3^. In conjunction with the observation of the increased circulating concentration of CXCL1, it is highly likely that the CXCL1-CXCR2 axis was involved in the focal accumulations of neutrophils that were found in IPAF lungs here and in other reports^8^,^20^,^21^,^22^,^23^,^24^,^25^.

A better understanding of the role that the CXCL1-CXCR2 axis plays in IPAF progression has many implications. First, the precise relationship of IPAF with other ILD is not known, despite the observation that IPAF and IIP may share similar histopathological patterns. However, the plasma level of CXCL1 in IPAF was observed to be significantly higher than that in IIP (Fig. 2A), which indicated that CXCL1 may help differentiate IPAF from IIP in some situations.

In addition, CXCL1 levels among the subjects with IPAF were inversely associated with DLCO (Fig. 3A) and positively correlated with FibMax scores and ESR levels (Fig. 3B and C). The IPAF patients who were experiencing AE at presentation or expected to have AE within the next 6 months also had significantly greater concentrations of CXCL1 in their plasmas (Fig. 4). However, no such correlations were found in IIP patients, which meant that CXCL1 levels here were relatively unique to IPAF, but not IIP. Therefore, CXCL1 can be used as a biomarker that could potentially identify those patients destined for more severe disease episodes and poor near-term prognoses.

More importantly, ILD is a chronic, progressive, and usually fatal pulmonary disease for which anti-inflammatory and immunosuppressive agents have been largely ineffective. Although these correlations do not imply any direct cause-effect relationships, the significant upregulation of CXCL1 observed in this group of patients with IPAF; the positive correlations between the CXCL1 concentration and FibMax, ESR, and BALF neutrophil counts as well as baseline CXCL1 concentration with acute exacerbations; and the reverse correlation between CXCL1 and DLCO might suggest a potential relevance of CXCL1 in the pathophysiology of IPAF. Furthermore, evidence already exists that inhibition of the CXCL1-CXCR2 pathway can control a variety of inflammatory conditions, such as rheumatoid arthritis^13^,^26^, inflammatory bowel disease^14^,^15^,^27^, acute respiratory distress syndrome^28^, pulmonary emphysema^16^, cystic fibrosis^6^,^17^, and chronic obstructive pulmonary disease^29^,^30^. Hence, the focus on the CXCL1-CXCR2 axis in IPAF may provide a novel approach to targeting up-stream processes in the progression of IPAF.

To our knowledge, this is the first study to suggest a possible role of CXCL1 and its cognate receptor CXCR2 in ILD. We observed extensive expression of CXCR2 in the leukocytes and endothelial cells in IPAF and IIP lungs (Fig. 5I,N,P,Q). Many manifestations ILD are characterized by the accumulation of inflammatory cells and vasculopathy within the lung, followed by the progressive deposition of extracellular matrix and the subsequent destruction of lung airspaces. CXCR2 is expressed in leukocytes, where its activation of CXCR2 induces a variety of cell responses, including degranulation, respiratory burst, phagocytosis, directed cell movement, integrin activation, and transmigration^31^. Similarly, among nonhaematopoietic cells, CXCR2 expression has been demonstrated in pulmonary endothelial cells^32^, where it has been associated with angiogenetic activity in lung tumours^9^ and fibroproliferative processes^33^. Thus, the contribution of CXCL1 and its receptor CXCR2 to the fibrotic process in IPAF should be further investigated.

In the present study, patients with IPAF were identified based on their radiologic or histopathologic patterns, including non-specific interstitial pneumonia (NSIP), organizing pneumonia (OP), NSIP with OP, and lymphoid interstitial pneumonia (LIP), according to the IPAF criteria^1^. Thus, in line with the inclusive criteria for IPAF, the IIP patients were recruited in our cohort based on the same requirements but without identifiable causes. The radiologic or histopathologic evidence of usual interstitial pneumonia (UIP) was not included as a specific morphologic feature because whether its presence alone in a patient with interstitial pneumonia can increase the likelihood of having an autoimmune condition remains a subject of debate^1^. However, patients with a radiologic/histopathologic UIP pattern are not excluded from the IPAF definition; therefore, individuals with UIP manifestation, including IPAF and idiopathic pulmonary fibrosis (IPF), should be investigated in a more inclusive cohort.

## Conclusion

In conclusion, our results provide new insights into the clinico-immunologic features of the recently proposed entity IPAF. Middle-age onset, an equal ratio of males to females, decreased DLCO, and elevated ESR were the typical characteristics of IPAF patients. Moreover, we highlight the role of the CXCL1-CXCR2 axis as a potential pathogenic mechanism closely associated with IPAF in which CXCL1-CXCR2 was likely involved in leukocyte recruitment and endothelial dysfunction. Taken together, our findings may contribute to the development of novel approaches for the treatment of this newly proposed manifestation of ILD.

## Methods

### Subjects and specimens

Plasma samples were available from 38 patients with IPAF, 81 patients with IIP, and 36 patients with chronic obstructive pulmonary disease (COPD), who were recruited from Huashan Hospital, Fudan University (Shanghai, China), between 1 January 2014 and 30 June 2015. Lung biopsy specimens were obtained from 5 patients with IPAF, 5 patients with IIP (obtained with diagnostic CT-guided lung needle biopsy) and 3 control patients (uninvolved tissue recovered during cancer resection surgery). All subjects with IPAF were retrospectively evaluated and fulfilled the 2015 European Respiratory Society (ERS)/American Thoracic Society (ATS) classification criteria for IPAF^1^. All subjects with IIP met the 2013 ATS/ERS Update of the International Multidisciplinary Classification of the IIP^34^, and had negative conventional autoimmune serologic tests. COPD was diagnosed by spirometry, and emphysema was detected and quantified by chest computed tomography scans^35^. Disease diagnoses were established by expert clinicians, who analyzed all information, and were masked to the experimental laboratory tests. Only the patients with radiologic or histopathologic manifestations of non-specific interstitial pneumonia (NSIP), organizing pneumonia (OP), NSIP with OP, or lymphoid interstitial pneumonia (LIP) in IPAF and IIP patients were included in this study. In patients with histological evidence, the diagnosis of interstitial pneumonia was dependent on pathological findings (i.e., surgical specimens or autopsy). In patients without histological evidence, the diagnosis was based on the findings of high-resolution computed tomography (HRCT) scans of the chest, medical history and physical examinations. Plasma, lung biopsies and bronchoalveolar lavage fluid (BALF) samples were obtained before the participants were given glucocorticoids or systemic immunosuppressants to avoid confounding effects of these medications on clinical parameters and cytokines expression.

Informed consent was obtained from each patient. This study was approved by the Ethics Committee of Huashan Hospital, Fudan University.

The methods were carried out in accordance with the approved guidelines and regulations. Additional methods are available in the Supplementary Methods section.

## Additional Information

**How to cite this article**: Liang, M. *et al*. Clinical Association of Chemokine (C-X-C motif) Ligand 1 (CXCL1) with Interstitial Pneumonia with Autoimmune Features (IPAF). *Sci. Rep.* **6**, 38949; doi: 10.1038/srep38949 (2016).

**Publisher’s note:** Springer Nature remains neutral with regard to jurisdictional claims in published maps and institutional affiliations.

## Supplementary Material

Supplementary Information

## Figures and Tables

**Table 1:** Demographic and Clinical Characteristics of the Lung Disease Subjects and Healthy Volunteers Who Had Plasma Cytokine Concentration Assays.

	IPAF	IIP	COPD	HC
N	38	81	36	101
Age, yr	56 ± 2.4	65 ± 1.7	70 ± 1.8	62 ± 2.4
Male	20(53)	52(64)	29(81)	56(55)
FVC, %predicted	69 ± 2.1	64 ± 1.8	83 ± 1.8	—
FEV1, %predicted	75 ± 2.4	71 ± 1.6	63 ± 2.4	—
FEV1/FVC	0.79 ± 0.017	0.79 ± 0.012	0.55 ± 0.012	—
DLCO, %predicted	46 ± 2.3	53 ± 1.3	58 ± 1.0	—

Data are presented as the means ± SE and in parentheses (median, minimum-to-maximum ranges). *IIP:* idiopathic interstitial pneumonia; *IPAF:* interstitial pneumonia with autoimmune features; *N:* number; *yr:* years; *FVC:* forced vital capacity; *FEV1:* forced expiratory volume in 1 second; *DLCO:* carbon monoxide diffusing capacity of the lung.

**Table 2:** Demographic and Clinical Characteristics of IPF Subjects Stratified by Plasma Chemokine (C-X-C motif) Ligan 1 Levels.

	Lowest CXCL1	Highest CXCL1	P
N	28	10	—
Age, yr	55 ± 2.6	52 ± 2.8	0.7871
Male	15(54%)	6(60%)	0.4509
FVC, % predicted	70 ± 2.6	68 ± 3.6	0.7241
FEV1, % predicted	78 ± 2.7	68 ± 4.7	0.0697
FEV1/FVC	0.80 ± 0.019	0.77 ± 0.038	0.4940
DLCO, %predicted	51 ± 2.3**	34 ± 2.9**	0.0003
FibMax	137.2 ± 14.7**	222.5 ± 28.4**	0.0069
ESR, mm/h	72.4 ± 3.3**	92.8 ± 5.2**	0.0023

Data are presented as the means ± SE and in parentheses (median, minimum-to-maximum ranges). *IIP:* idiopathic interstitial pneumonia; *IPAF:* interstitial pneumonia with autoimmune features; *N:* number; *yr:* years; *FVC:* forced vital capacity; *FEV1:* forced expiratory volume in 1 second; *DLCO:* carbon monoxide diffusing capacity of the lung; *ESR:* erythrocyte sedimentation rate.
