# Supplementary material for: Clinical Association of Chemokine (C-X-C motif) Ligand 1 (CXCL1) with Interstitial Pneumonia with Autoimmune Features (IPAF)
Source: Sci Rep. 2016 Dec 13;6:38949. doi: 10.1038/srep38949 (PMC5154180; doi:10.1038/srep38949)
Supplement: Supplementary Information [file srep38949-s1.docx]

**Clinical Association of Chemokine (C-X-C motif) Ligand 1 (CXCL1) with Interstitial Pneumonia with Autoimmune Features (IPAF)**

**Minrui Liang^1,2,5^, Zhixing Jiang^1,2,5^, Qiong Huang^3^, LeiLiu^1,2^, Yu Xue^1,2^, Xiaoxia Zhu^1,2^, Yiyun Yu^1,2^, Weiguo Wan^1,2^, *Haihua Yang^4^, *Hejian Zou^1,2^,**

**Author affiliations**

^1^Division of Rheumatology, Huashan Hospital, Fudan University, Shanghai 200040, PR China. ^2^Institute of Rheumatology, Immunology and Allergy, Fudan University, Shanghai 200040, PR China. ^3^Department of Dermatology, Huashan Hospital, Fudan University, Shanghai 200040, PR China. ^4^Department of Pulmonology, Huashan Hospital, Fudan University, Shanghai 200040, PR China. ^5^These authors contributed equally to this work. *Correspondence: Dr HJ Zou, MD, PhD, Division of Rheumatology, Huashan Hospital, Fudan

University, 12 No. Wulumuqi Zhong Road, Shanghai 200040, China hjzou@fudan.edu.cn, or Dr HH Yang, MD, Department of Pulmonology, Huashan Hospital, Fudan University, 12 No. [Wulumuqi Zhong Road, Shanghai 200040, China, haihuayouxiang@163.com.](mailto:haihuayouxiang@163.com)

**Supplementary Information**

**Supplementary Methods**

**Supplementary Methods Bronchoalveolar lavage fluid (BALF)**

Six patients with IPAF and 6 patients with IIP underwent bronchoalveolar lavage according to accepted international guidelines^1-3^. The sampling area was determined on the basis of the lesion location on chest HRCT images, which were recorded within 1 week of lavage. BALF was performed with a total volume of 200 ml sterile 0.9% saline (4×50 ml aliquots), which was manually aspirated and returned together and separated into four aliquots. The first fraction was used for Gram staining, acid-fast staining, calcofluor white fungal staining, and aerobic bacterial, fungal, and mycobacterial cultures. The second fraction was filtered through coarse surgical gauze to remove mucous debris and centrifuged at 500×g for 6 min at 4°C and used for malignant cell cytology. The third fraction was processed as the second fraction and was also used for cytology assessment. A cell count was performed using a haemocytometer, and the cell differential counts were analysed following Giemsa staining of a cytospin slide. Cell counts performed on the BALF fluid included macrophage, lymphocyte, neutrophil, and eosinophil cell counts. The supernatant of the fourth fraction was collected and frozen at -80°C for subsequent cytokine analysis.

**Pulmonary function tests (PFTs)**

The following parameters concerning pulmonary function were evaluated by spirometry: forced vital capacity (FVC), forced expiratory volume in 1 s (FEV1), the FEV1/FVC ratio and the diffusing capacity of the lung for carbon monoxide (DLCO). PFTs were performed as part of the enrolment protocol, and the findings were assessed according to American Thoracic Society recommendations^4^.

1

**HRCT**

All IPAF and IIP patients underwent HRCT of the chest. Scans were read by two specialists (HH Yang and L Liu, Huashan Hospital) with an interest in interstitial lung disease who were blinded to the study conditions. Briefly, as described previously^5-6^, each lung was divided into the following 3 zones: upper (lung apex to aortic arch), middle (aortic arch to the inferior pulmonary veins), and lower (inferior pulmonary veins to the lung bases). For each HRCT scan, the extent of the pulmonary abnormality in each of the 6 zones was calculated. The sum of all scores in the 6 lung zones is referred to as the FibMax. The score was based on the percentage of the lung parenchyma that showed evidence of the abnormality and was estimated to the nearest 5% of parenchymal involvement^5,6^.

**Circulating and BALF cytokines**

The concentrations of CXCL1, IL-4, IL-13, IL-6, IL-17, IFN-γ, CXCL2, CXCL5, and CXCL8 were measured in plasma and BALF specimens with a ProcartaPlex Human panel (eBioscience, San Diego, California, USA) in a Luminex system (Magpix, Luminex, Austin, Texas, USA) according to the manufacturer’s instructions.

**Histological analysis**

The specimens were fixed in 10% neutral buffered formalin solution and embedded in paraffin. Multiple 5-µm sections were stained with haematoxylin and eosin (H&E) for histopathological analysis. Cell subtypes visualized by immunohistochemistry using antibodies specific for CD45, CXCR2, and MPO were assessed on deparaffinized human lung sections. Fibroblasts were identified by staining with α-SMA. These primary monoclonal antibodies (mAbs) were all obtained from Abcam (Cambridge, MA, USA). Briefly, tissue sections were rehydrated, rinsed with citrate buffer, blocked and washed with Tris-buffered saline Tween-20 (TBST), and incubated with primary antibodies. After being washed, the tissue sections were incubated with horseradish peroxidase-conjugated goat anti-rat/rabbit IgG (Dako, Glostrup, Denmark) secondary antibody. After development with 3,3’­diaminobenzidine tetrahydrochloride and hydrogen peroxide, the sections were counterstained with haematoxylin. H&E staining and comparative immunohistochemical analysis for CD45, α-SMA, CXCR2, and MPOwere performed on serial sections.

**Statistical analysis**

The significance of differences in the baseline clinical and demographic features was determined with a univariate analysis using Fisher’s exact test (for binary and categorical variables) or Wilcoxon’s rank sum test (for continuous variables). Comparisons of the cytokine concentrations were analysed with a one-way analysis of variance (ANOVA) with a Bonferroni adjustment for multiple comparisons. Two-group comparisons were analysed with Student’s t-test. Correlations were tested and presented as Pearson’s correlation coefficient (*r*) or as the coefficient of correlation (*r^2^*). Unless otherwise noted, the data are presented as the *means±SE* and in parentheses (median, minimum-to-maximum ranges).

**Reference:**

1 Haslam, P. L. & Baughman, R. P. Report of ERS Task Force: guidelines for measurement of acellular components and standardization of BAL. *The European respiratory journal* **14**, 245-248 (1999).

2 Meyer, K. C. *et al.* An official American Thoracic Society clinical practice guideline: the clinical utility of bronchoalveolar lavage cellular analysis in interstitial lung disease. *American journal of respiratory and critical care medicine* **185**, 1004-1014, doi:10.1164/rccm.201202-0320ST (2012).

3 Technical recommendations and guidelines for bronchoalveolar lavage (BAL). Report of the European Society of Pneumology Task Group. *The European respiratory journal* **2**, 561-585 (1989).

4 Lung function testing: selection of reference values and interpretative strategies. American Thoracic Society. *The American review of respiratory disease* **144**, 1202-1218, doi:10.1164/ajrccm/144.5.1202 (1991).

5 Zou, J., Guo, Q., Chi, J., Wu, H. & Bao, C. HRCT score and serum ferritin level are factors associated to the 1-year mortality of acute interstitial lung disease in clinically amyopathic dermatomyositis patients. *Clinical rheumatology* **34**, 707-714, doi:10.1007/s10067-015-2866-5 (2015).

6 Goldin, J. G. *et al.* High-resolution CT scan findings in patients with symptomatic scleroderma-related interstitial lung disease. *Chest* **134**, 358-367, doi:10.1378/chest.07-2444 (2008).

**Supplementary Figures**

**Supplementary Figure 1.**

**Supplementary Figure 1.** Concentrations of circulating CXCL2 (A), CXCL5 (B), and CXCL8 (C) in the plasma of lung disease subjects using a multiplex Luminex immunoassay. The horizontal line from bottom to top denotes the minimum, 25th percentile, median, 75th percentile, and maximum. Mean values are denoted by “+”. *P<0.05 and **P<0.01 compared with the other cohort.


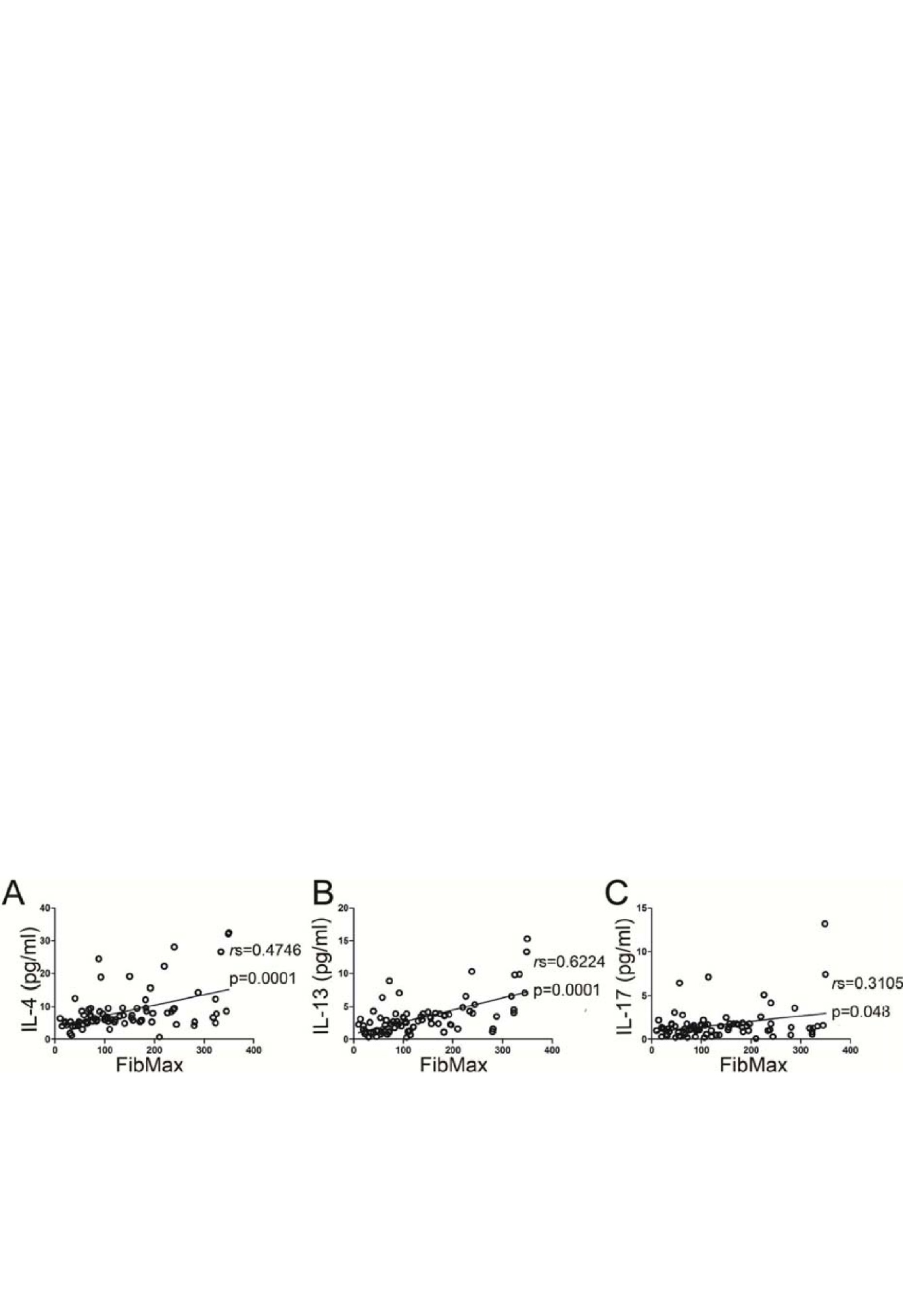


**Supplementary Figure 2**.

**Supplementary Figure 2.** Clinical correlations of circulating cytokine levels in IIP patients. The only statistically significant correlations in subjects with IIP between circulating IL-4, IL-13, IL-6, IL-17, INF-γ levels and the cross-sectional demographic or pulmonary function/radiographic characteristics at the time of specimen acquisitions were positive associations of IL-4 (A), IL-13 (B) or IL-17 (C) with FibMax score.

**Supplementary Figure 3.**


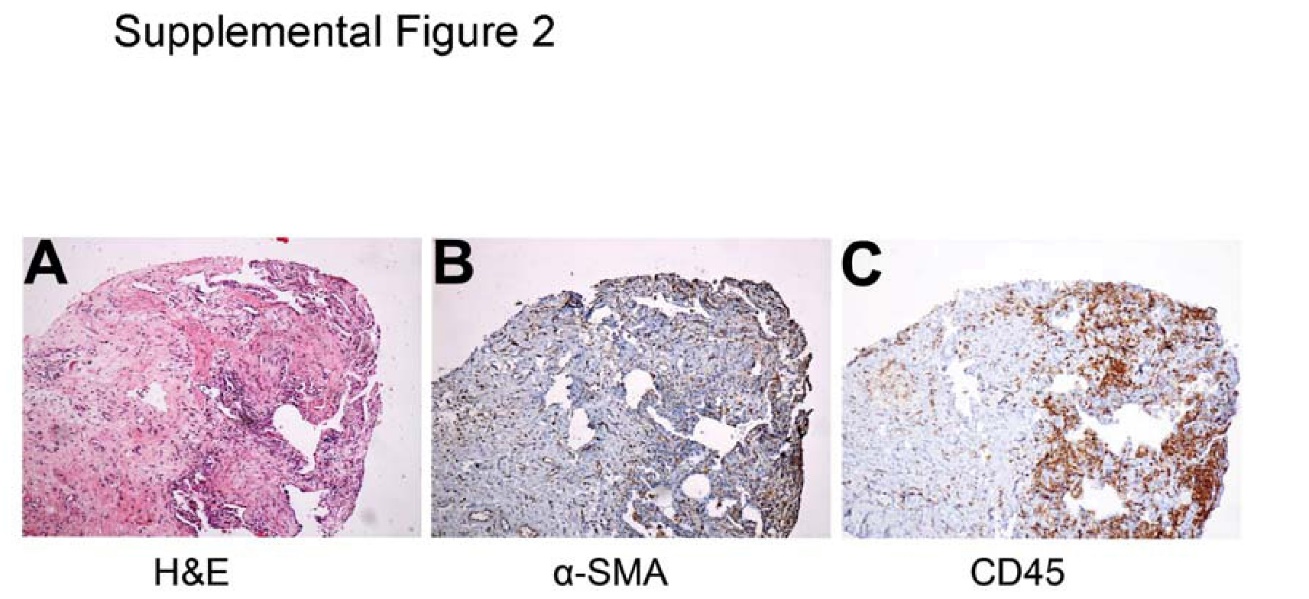


**Supplementary Figure 3.** Haematoxylin and eosin (H&E) staining (A) and immunohistochemical staining for α-SMA (B) and CD45 (C) were performed on the lung needle biopsies of an IPAF patient. CD45^+^ immunoreactive leukocytes were typically observed near α-SMA^+^ fibroblast foci in IPAF lungs. (A-C: for original magnification×100).

**Supplementary Figure 4.**

**Supplementary Figure 4.** Concentrations of CXCL1, CXCL2, CXCL5, and CXCL8 in bronchoalveolar lavage fluid (BALF) of lung disease subjects using a multiplex Luminex immunoassay. The horizontal line from bottom to top denotes the minimum, 25th percentile, median, 75th percentile, and maximum. Mean values are denoted by “+”. *P<0.05 and **P<0.01 compared with the other cohort.

| **Supplementary Tables** | |  |  |  |  |
| --- | --- | --- | --- | --- | --- |
|  | **Supplementary Table 1. Demographic and Clinical Characteristics of Lung Disease** | | |  |  |
|  | **Subjects Who Underwen Bronchoalveolar Lavage Fluid Cytokine Concentration Assays** | | |  |  |
|  |  | **IPAF** | **IIP** | **P** | |
|  |  |  |  |  |  |
|  | **N** | 6 | 6 | - |  |
|  | **Age, yr** | 49±6.9* | 67±3.1* | 0.0447 |  |
|  | **Male** | 3(50%) | 5(80%) | 0.2727 |  |
|  | **FVC, % predicted** | 75±5.5 | 71±7.1 | 0.6771 |  |
|  | **FEV1, % predicted** | 72±4.3 | 68±4.6 | 0.5219 |  |
|  | **FEV1/FVC** | 0.78±0.023 | 0.80±0.050 | 0.7647 |  |
|  | **DLCO, % predicted** | 49±4.0 | 52±6.9 | 0.6855 |  |
|  | Data are presented as the means±SE and in parentheses (median, minimum-to-maximum ranges). *IIP:* idiopathic interstitial pneumonia; *IPAF*: interstitial pneumonia with autoimmune features; *N:* number; *yr:* years; *FVC:* forced vital capacity; *FEV1:* forced expiratory volume in 1 second; *DLCO:* carbon monoxide diffusing capacity of the lung. | | | |  |

| **Supplementary Table 2. Cell Counts in the Bronchoalveolar Lavage Fluid of Subjects with Interstitia Pneumonia with Autoimmune Features and with Idiopathic Interstitial Pneumonia.** | | | |
| --- | --- | --- | --- |
|  | **IPAF** | **IIP** | **P** |
| **Neutrophils, % in BALF** | 29.3±3.7* | 16.5±3.7* | 0.0341 |
| **Lymphocytes, % in BALF** | 7.7±3.9 | 12.2±2.0 | 0.3263 |
| **Macrophages, % in BALF** | 63.3±5.6 | 70.7±4.5 | 0.3327 |
| **Eosinophils, %in BALF** | 0.5±0.3 | 0.7±0.3 | 0.7342 |
| Data are presented as the means±SE and in parentheses (median, minimum-to-maximum ranges). *IIP:* idiopathic interstitial pneumonia; *IPAF:* interstitial pneumonia with autoimmune features; *BALF:* bronchoalveolar lavage fluid. | | | |

**Abbreviations**

interstitial pneumonia with autoimmune features(IPAF)

idiopathic interstitial pneumonia (IIP)

chemokine (C-X-C motif) ligand 1(CXCL1)

diffusing capacity of the lungs for carbon monoxide (DLCO)

erythrocyte sedimentation rate (ESR) chronic obstructive pulmonary diseases (COPD) bronchoalveolarlavage fluid (BALF)

interleukin (IL)

chemokine (C-X-C motif) receptor 2 (CXCR2)

interferon-γ(IFN-γ)

myeloperoxidase（MPO）
